# Summarizing cellular responses as biological process networks

**DOI:** 10.1186/1752-0509-7-68

**Published:** 2013-07-29

**Authors:** Christopher D Lasher, Padmavathy Rajagopalan, T M Murali

**Affiliations:** 1 Genetics, Bioinformatics, and Computational Biology Ph.D. Program, Virginia Tech, Blacksburg, VA 24061 USA; 2 Department of Chemical Engineering, Virginia Tech, Blacksburg, VA 24061 USA; 3 ICTAS Center for Systems Biology of Engineered Tissues, Virginia Tech, Blacksburg, VA 24061 USA; 4 Department of Computer Science, Virginia Tech, Blacksburg, VA 24061 USA

**Keywords:** Molecular interaction networks, Gene expression data, Networks of biological processes, Data integration, Markov chain Monte Carlo

## Abstract

**Background:**

Microarray experiments can simultaneously identify thousands of genes that show significant perturbation in expression between two experimental conditions. Response networks, computed through the integration of gene interaction networks with expression perturbation data, may themselves contain tens of thousands of interactions. Gene set enrichment has become standard for summarizing the results of these analyses in terms functionally coherent collections of genes such as biological processes. However, even these methods can yield hundreds of enriched functions that may overlap considerably.

**Results:**

We describe a new technique called Markov chain Monte Carlo Biological Process Networks (MCMC-BPN) capable of reporting a highly non-redundant set of links between processes that describe the molecular interactions that are perturbed under a specific biological context. Each link in the BPN represents the perturbed interactions that serve as the interfaces between the two processes connected by the link.

We apply MCMC-BPN to publicly available liver-related datasets to demonstrate that the networks formed by the most probable inter-process links reported by MCMC-BPN show high relevance to each biological condition. We show that MCMC-BPN’s ability to discern the few key links from in a very large solution space by comparing results from two other methods for detecting inter-process links.

**Conclusions:**

MCMC-BPN is successful in using few inter-process links to explain as many of the perturbed gene-gene interactions as possible. Thereby, BPNs summarize the important biological trends within a response network by reporting a digestible number of inter-process links that can be explored in greater detail.

## Background

### Motivation

The deluge of publicly available molecular biology data, including genome-wide gene expression measurements [1,2] and gene and protein interaction networks [3,4] has necessitated the development of computational methods that produce comprehensible views of large numbers of biological molecules and their connections. Reporting perturbation in gene expression on the basis of individual genes [5,6] (of which there may be thousands) has given way to more holistic techniques—referred to as functional enrichment—that instead report the significance of the collective perturbation of processes—sets of biologically related genes (e.g., [7-11]. Results from these analyses reveal important trends that large lists of genes can obscure.

Recent work in functional enrichment of gene expression data [9,11] has emphasized finding concise, non-redundant sets of processes that account for much of the overall perturbation among the genes. Methods by Lu *et al.*[9] and Bauer, Gagneur, and Robinson [11] assume that a cell or tissue perturbs certain biological processes in response to a change internal or external conditions. In their models, perturbed processes cause the perturbation of the expression of individual genes belonging to each of those processes. They consider the actual measurements of perturbation of the individual genes, as assessed by DNA microarrays, for example, as noisy observations of signals generated by perturbation of specific processes by cells in response to a stimulus. Both methods use generative models to assess the goodness of fit of a set of candidate perturbed processes to the observed gene perturbations, while differing in their precise formulations. The two methods use standard algorithms (greedy [9] and Markov chain Monte Carlo (MCMC) [11]) to find the set of processes with the greatest fit to the observed data.

Products of genes do not act independently but rather in concert with products of other genes through numerous interactions. In a similar vein, biological processes, composed of genes, may themselves interact. Accordingly, researchers have begun developing methods to identify connections between processes based on the underlying gene interaction networks. A method by Li *et al.*[12] computed the “crosstalk” between processes by counting the number of interactions that occur among the genes of each process and assessing the significance of this number against empirical distributions. Dotan-Cohen *et al.* published a more direct method [13] which uses Fisher’s Exact Test to determine if one process is linked to another, i.e., if genes in the first process have significantly more interactions with genes in the second process than would be expected by chance. Wang *et al.*[14] published a method that calculates what they call “functional similarity” between two processes using the sum of the distances between all pairs of genes belonging to those processes.

While the previous methods represent advances in finding high-level connections between processes, they do not incorporate information which could lead to discovering which connections have relevance under specific biological contexts. Motivated by this methodological gap, in earlier work [15] we extended the method of Dotan-Cohen *et al.*[13] by integrating gene expression data with gene-gene interactions to compute what we termed “Contextual Biological Process Linkage Networks” (CBPLNs). A link in a CBPLN indicates not only that the genes of two processes have a significant number of interactions among them, but that genes at the interface exhibit a large amount of perturbation in expression. Thus, it became possible to infer the inter-process connections relevant to a cell or tissue’s response to an internal or external stimulus.

The CBPLN method has several aspects that need improvement. First, because it must build empirical distributions to determine the significance of each link, it becomes prohibitively computationally expensive as the number of links to test grows. Second, the method reports all significant links, Since it makes no distinction among two or more links that are found to be significant on account of nearly identical sets of gene-gene interactions, it may output many redundant significant links. This latter drawback is universal to all methods that compute inter-process links, and also to most techniques for functional enrichment.

Here we present a new method that simultaneously addresses the shortcomings of earlier methods. Our method takes inspiration from the methods for functional enrichment reported by Lu *et al.*[9] and Bauer *et al.*[11]. We assume that links between biological processes become perturbed during the response of a cell or tissue to some stimulus, and that this perturbation of inter-process links propagates via the individual gene-gene interactions between genes belonging to the different processes. We can not directly observe perturbation of the links between the processes; instead, our method considers the perturbation of genes participating in the interfacing interactions of processes as noisy observations generated from the perturbed inter-process links. Our method infers a non-redundant set of processes and their perturbed links, which we call a Biological Process Network (BPN), from the interactions between the observed perturbed genes. We compute the likelihood of candidate BPNs in terms of parameters accounting for the noisiness in the observed states of the gene-gene interactions. Using Markov chain Monte Carlo (MCMC), we identify BPNs of high likelihood. We label this new method “MCMC Biological Process Networks” (MCMC-BPN). BPNs thus computed summarize the important biological trends within a response network by reporting to the user a digestible number of inter-process links that can be explored in greater detail.

### Overview of the method

MCMC-BPN aims to explain as many interactions between genes with perturbed expression by as few inter-process links as possible. By including a link between a pair of processes in the BPN, we say that link “explains” the interactions cross-annotated by that pair of terms. Our objective is to the reward inclusion of links in the BPN that explain many interactions between perturbed genes not already explained by other links in the BPN. Simultaneously, another objective is to penalize the inclusion of more links in the BPN than necessary, including links which mostly explain unperturbed interactions, and missing a large number of perturbed interactions. To this end, we define a likelihood function as the product of several Bernoulli distributions, controlled by three parameters used to adjust for the amount of “noise” in the observed perturbation of the cross-annotated links.

The first of these parameters, the link prior *λ*, serves to reduce the number of links in a BPN, for when *λ* is low, having few links increases the overall likelihood. A low value for the second parameter, the false-positive rate *α*, encourages BPNs that explain many perturbed interactions. Finally, when the parameter *β*, which represents the false-negative rate, has a low value, it encourages BPNs that explain few unperturbed interactions. Note that modifying the BPN in such a way that increases the contribution of one parameter to the likelihood may be offset, or even outweighed, by a decrease in the contribution to the likelihood by another parameter. For example, including every possible link in a BPN will have a favorable likelihood contribution via *α*, since it necessarily explains all perturbed interactions. Such an inclusive BPN will, however, lead to very poor contributions via *λ* (many links are included in the BPN) and *β* (many unperturbed interactions will also be included). Thus, such a BPN will have a very low overall likelihood. A desirable BPN must strike a balance among the tension of all three parameters—neither including too many links, nor explaining too few perturbed interactions, nor explaining too many unperturbed interactions—in order maximize the overall likelihood.

While the likelihood function provides a means of scoring the quality of a given BPN, for any given data set, there exist 2^|*L*|^ possible BPNs, where *L* is the set of all possible links between pairs of processes. To search this potentially enormous solution space, we use the Metropolis-Hastings algorithm for Markov chain Monte Carlo (MCMC) [16]. Each state in the Markov chain represents a particular set of values for the parameters *λ*, *α*, and *β*, as well as a particular configuration of inter-process links. The neighbors of the state are those which have one additional or one less link, or which have one parameter with a different value. The parameter values and links which contribute to BPNs with high likelihoods will tend to remain consistent from one visited state to the next. Thus, we report the final BPN as the links that appear most frequently throughout the states visited during the MCMC.

### Application

We applied MCMC-BPN to three treatment-control experiments relating to the liver and liver disease. In the first application, we compared gene expression of rat hepatocytes in two common *in vitro* culture systems [17]: hepatocyte monolayer (HM) and collagen sandwich (CS). The remaining two experiments contrast gene expression levels from liver tissue samples from dozens of human patients diagnosed with hepatitis C virus (HCV)-induced cirrhosis and hepatocellular carcinoma (HCC) with expression levels of samples from healthy patients [18]. Approximately 170 million people worldwide suffer from HCV infection [19]. HCC ranks third among the deadliest cancers worldwide, of which HCV is among the leading causes of incidence [20]. Below, we present and discuss the BPNs computed to summarize the major trends of differential expression of each of these three data sets. We found the BPNs contained links between biological processes that were anticipated, as well as unexpected connections that suggest further exploration.

## Results

### Data sources and contrasts

Table 1 summarizes the data sources for the three contrasts we studied. For the “CS vs. HM” contrast, we used the samples for CS day 8 as the treatment and samples for HM day 8 as the control. For this contrast, we pruned the STRING network to include those interactions with a score of 500 or greater. For the “Cirrhosis” contrast, we used samples from patients designated to be in the cirrhosis category as the treatment; for the “Very Advanced HCC” contrast, we used samples from patients designated as being in the “Very Advanced HCC” category as the treatment; in both contrasts, we used the samples from uninfected patients as the control.

**Table 1:** Data sources for each contrast

**Contrast**	**Organism**	**Sample**	**database** **Interactions**	**series** **GEO**
CS vs. HM [17]	*Rattus norvegicus*	Hepatocyte	STRING v8.3 [4]	GSE20659
		Culture		
Cirrhosis [18]	*Homo sapiens*	Liver	MiMI [3]	GSE6764
		Biopsy		
Very Advanced HCC [18]	*Homo sapiens*	Liver	MiMI [3]	GSE67674
		Biopsy		

We obtained functional annotations for the genes from the c2 canonical pathways and c5 GO gene sets of the Molecular Signatures Database (MSigDB) version 3.0 [7], downloaded on February 7, 2011, CORUM complexes [21] downloaded on February 7, 2011, NetPath signal transduction pathways [22] downloaded June 6, 2009, and NCI Pathway Interaction Database’s curated pathways [23] downloaded February 7, 2011. For the rat data, we normalized all data into the Ensembl Peptide ID namespace through a combination of the Synergizer [24] and MadGENE [25] mapping services. For the human data, we used the same services to normalize all the data into Entrez Gene namespace.

Next, we integrated the annotations with the gene interaction networks. We say that a pair of processes “cross-annotates” interactions in the underlying gene-gene interaction network if one of the two genes belongs to one of the processes in the link and the other gene belongs to the other process. (See the section titled “The MCMC-BPN algorithm” for details.) For each contrast, Table 2 presents the number of processes, the number of cross-annotating pairs among these processes, the number of interactions which the process pairs cross-annotate, and the number of those interactions which we consider “perturbed” (i.e., both interacting genes exhibit perturbed expression for that contrast; see the section titled “The MCMC-BPN algorithm” for details).

**Table 2:** Statistics on inputs by contrast

**Contrast**	**Processes**	**process pairs** **Cross-annotating**	**interactions** **Cross-annotated**	**interactions** **Perturbed**
CS vs. HM	210	14796	11585	3861
Cirrhosis	148	8714	12913	1954
Very Advanced HCC	345	36460	30201	15400

For each contrast, we performed a total of five runs of MCMC-BPN. Each run took between 15 and 30 hours on a single core of a 2.8GHz AMD Opteron 4184 processor using our implementation in Python. We first describe results on the consistency of the BPNs computed by the different MCMC runs and summarize BPN statistics. Second, we show that the BPNs contain very little redundancy. Third, for each contrast, we display an example BPN and provide detail on several interesting links in the reported BPNs. Fourth, we demonstrate that the BPNs produced by MCMC-BPN are more informative while also being less redundant than those computed by two previous methods: CBPLN [15] and biological process linkage networks (BPLN) [13]. Finally, we describe some general observations of the behavior of the MCMC and features which affect the performance of our algorithm. Two additional files accompany these results. The supplementary information (Additional file 1) contains (a) details on how we executed the MCMC-BPN software to obtain and visualize our results and (b) a description of the files in the supplementary results, which are available in Additional file 2. This file contains all the five BPNs for each of the contrasts studied and the parameters estimated by each run of the software.

### Consistency and statistics of BPNs computed by MCMC-BPN

**Figure 1:**
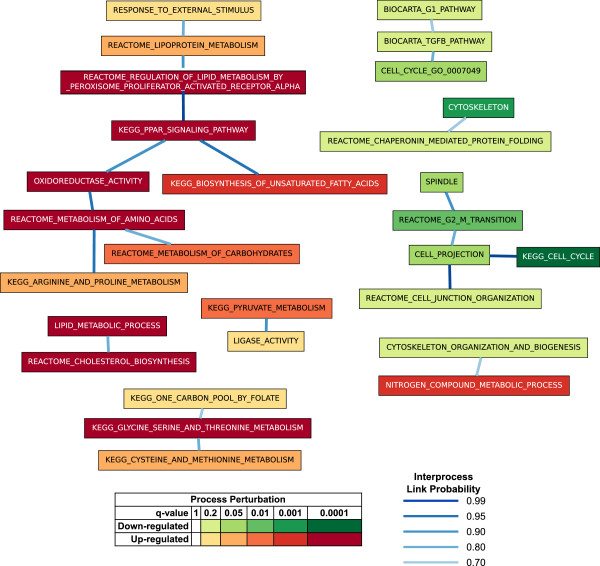
**Pairwise overlaps of BPNs for the three contrasts.** Heatmaps for each contrast, CS vs. HM, Cirrhosis, and Very Advanced HCC, indicate the pairwise overlap of perturbed interactions (top), and unperturbed interactions (bottom) explained by the BPNs.

We measured the consistency between the five BPNs for each contrast in two ways: how many links (i.e., the pairs of processes) each pair of BPNs shared, and how many explained interactions each pair of BPNs had in common. The average Jaccard Index (JI) for all ten pairwise comparisons of the shared links in the CS vs. HM BPNs was 0.91; in these and subsequent results, we report averages but not the standard deviations, since they were one to two orders of magnitude smaller than the averages. Figure 1 presents, for each of the three contrasts, a pair of heatmaps showing the overlap between each pair of BPNs on the basis of their common perturbed and unperturbed explained interactions. For the CS vs. HM contrast, the average Jaccard Index for the common perturbed interactions was 0.92, illustrating the high degree of overlap between the reported BPNs. The five BPNs for the CS vs. HM contrast consisted of an average of 27.6 processes with 20.0 inter-process links explaining 1686.0 interactions, of which 1070.2 interactions were perturbed. The BPNs explained 27.7% of all perturbed interactions using 0.1% of the possible links.

Unlike the CS vs. HM contrast, the BPNs reported for the Cirrhosis contrast showed mixed consistency. Figure 1 (center) clearly illustrates the divergence in the BPNs computed for the Cirrhosis contrast in terms of the overlap of their explained interactions. Three of the five BPNs (BPNs 1, 3, and 4) were identical, with 18 processes and 14 links between these processes. The two remaining BPNs had only two processes with one link and four processes with two links, respectively, none of which were present in the three identical BPNs. We found that *β*, the false-negative rate, took on a very high value (0.95) for these runs in comparison to the others (0.6). This value of *β* indicated that only 5% of the interactions explained by these BPNs were perturbed. We discarded these two BPNs from further analyses, reasoning that they represented a situation where the MCMC could not escape a local minimum. We found that the 14 links of the three remaining BPNs explained 947 interactions, 380 of which were perturbed. Thus the BPNs explained 19.5% of all perturbed interactions using 0.2% of all possible links.

Similar to the Cirrhosis contrast, three of the BPNs computed for the Very Advanced HCC contrast had a high degree of similarity (BPNs 1, 2, and 4 in Figure 1 (right)). The remaining two BPNs, which had a modest similarity to each other, showed very little overlap with the first three BPNs. Unlike the Cirrhosis contrast, the two groups of BPNs had similar numbers of processes and links; the three similar BPNs had a mean of 44.0 processes with 36.7 links between them, and the two remaining BPNs had a mean of 38.5 processes and 41.5 links. They differed remarkably, however, in the number of interactions their links explained. The three similar BPNs explained a mean of 8114.3 interactions, of which 5670.7 were perturbed. The remaining two BPNs explained a mean of 3470.5 interactions, of which 890.5 were perturbed. As in the Cirrhosis contrast, we found that *β* assumed high values (0.7 and 0.75) in these two runs compared to the others (0.3). Again reasoning the MCMC may have failed to escape local minima, we excluded the two dissimilar BPNs from the remainder of our analyses.

### Lack of redundancy in BPNs

We sought to determine whether there was any redundancy within each BPN for each contrast. We used two measures for this evaluation: (i) the overlap among links in a BPN in terms of common interactions and (ii) the number of links in each BPN that explained each interaction. We define these measures in more detail in the section titled “ Measuring redundancy within a BPN.”

**Figure 2:**
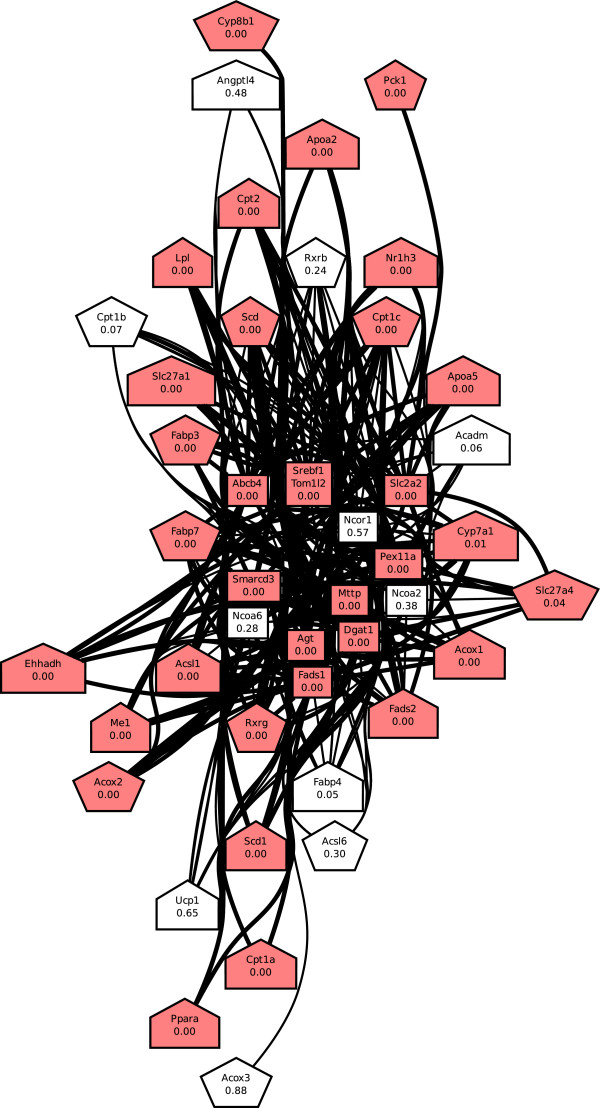
**Redundancy of links within BPNs.** In each plot, the *x*-axis corresponds to the maximum observed JI for a given link, while the *y*-axis corresponds to the percentage of links with a maximum observed JI within each corresponding bin on the *x*-axis. The height of the bar indicates the average over the BPNs analyzed for each contrast. Error bars represent one standard deviation from the mean. For each contrast, histograms show the results when considering perturbed interactions (top) and unperturbed interactions (bottom).

We measured the amount of overlap between every pair of links within each BPN in terms of the number of common explained interactions, averaging the results over the BPNs computed for each contrast. Figure 2 displays, for each contrast, the distributions of the maximum observed JIs for each link, divided into perturbed explained interactions and unperturbed explained interactions. Among the five CS vs. HM BPNs, when considering perturbed interactions, a mean of 80.0% of links had a maximum JI between 0 and 0.2. For unperturbed interactions, this number was 59.9%. Moreover, 80.7% of perturbed explained interactions and 82.2% of unperturbed explained interactions in CS vs. HM had only one link explaining them on average.

Links in Cirrhosis BPNs also exhibited little overlap (see Figure 2 (center)), with all links having a maximum JI of at most 0.2, both for perturbed and for unperturbed interactions. At least 85% of the perturbed explained interactions and the unperturbed explained interactions were explained by only one link. Links exhibited little overlap in Very Advanced HCC BPNs as well, as shown in Figure 2 (right). Nearly 90% of the links had a maximum JI of at most 0.2 in the case of perturbed explained interactions, with the number being nearly 70% for unperturbed explained interactions. Moreover, about 72% of both perturbed and unperturbed explained interactions were explained by only one link.

Overall, the dominance of low JIs for the processes and links indicated that the BPNs computed by MCMC-BPN demonstrated very little redundancy. The fact that most explained interactions had only one explaining link supported this observation.

### Interpretation of the BPNs

#### CS vs. HM

**Figure 3:**
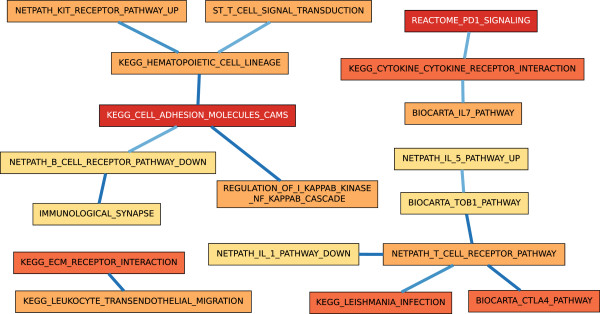
**A BPN computed for the CS vs HM contrast.** Rectangular nodes represent biological processes. Node color indicates collective perturbation of the process, as assessed by GSEA, where deep reds signify significant collective up-regulation, and deep greens signify collective down-regulation. Edges between process nodes represent significant links in the BPN. Dark blue edges indicate highly probable links, while light blue edges indicate less probable links.

Figure 3 presents one of the BPNs computed using the MCMC-BPN method on the data for the CS vs. HM contrast. The BPN contained up- and down-regulated processes in different components. Most up-regulated processes were related to metabolic functions performed by the liver, including lipid and carbohydrate metabolism, while most down-regulated processes related to cell replication and the cytoskeleton. These reflect the greater retention of physiological function of hepatocytes in CS culture versus HM culture, and the greater degree of de-differentiation for cells in HM versus CS, respectively, as reported by Kim *et al.*[17].

Two main components dominate the BPNs. The first component contained a mix of processes related to fatty acid metabolism (OXIDOREDUCTASE_ACTIVITY, KEGG_PPAR_SIGNALING_PATHWAY, REACTOME_ REGULATION_OF_LIPID_METABOLISM_BY_PEROXISOME_PROLIFERATOR_ACTIVATED_RECEPTOR_ ALPHA, and KEGG_BIOSYNTHESIS_OF_UNSATURATED_FATTY_ACIDS) and processes related to amino acid and carbohydrate metabolism (REACTOME_ METABOLISM_OF_CARBOHYDRATES, REACTOME_ METABOLISM_OF_AMINO_ACIDS, and KEGG_ARGININE_AND_PROLINE_METABOLISM), all critical functions carried out by hepatocytes [26]. A link between OXIDOREDUCTASE_ACTIVITY and REACTOME_METABOLISM_OF_AMINO_ACIDS bridges these two groups of processes. The second component contained down-regulated processes related to the de-differentiation of the hepatocytes in HMs.

**Figure 4:**
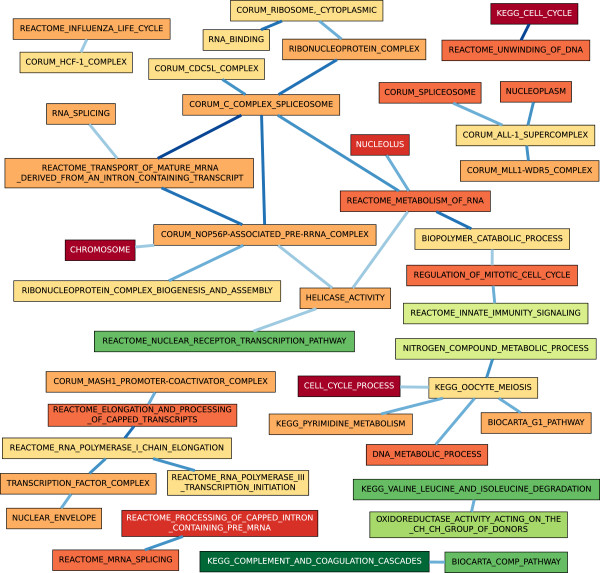
**Interactions explained by the link between two PPAR-related processes.** Pentagonal nodes represent genes belonging to KEGG_PPAR_SIGNALING_PATHWAY, rectangular nodes represent genes belonging to REACTOME_REGULATION_OF_LIPID_METABOLISM_BY_PEROXISOME_PROLIFERATOR_ACTIVATED_RECEPTOR_ALPHA, and house-shaped nodes represent genes belonging to both processes. Red-colored nodes indicate perturbed genes. Edges between nodes represent gene-gene interactions, with a bold edge representing a perturbed interaction.

Although the names of some of the processes appear to be very similar, their actual gene content tended to overlap very little. For example, the sets of genes annotated to CELL_CYCLE_GO_0007049 and to KEGG_CELL_CYCLE had JI of only 0.23. Similarly KEGG_PPAR_SIGNALING_PATHWAY and REACTOME_REGULATION_OF_LIPID_METABOLISM_BY_PEROXISOME_PROLIFERATOR_ACTIVATED_RECEPTOR_ALPHA, which are directly linked in the BPN, had a genes-based JI of 0.32. Figure 4 shows the dense network of interactions explained by this link. While genes belonging to both processes, such as peroxisome proliferator-activated receptor *α*(PPARA) and cholesterol 7 *α*-hydroxylase (CYP7A1), are involved in some interactions, there are many interactions that involve genes belonging to only one of the two processes.

#### Cirrhosis

**Figure 5:**
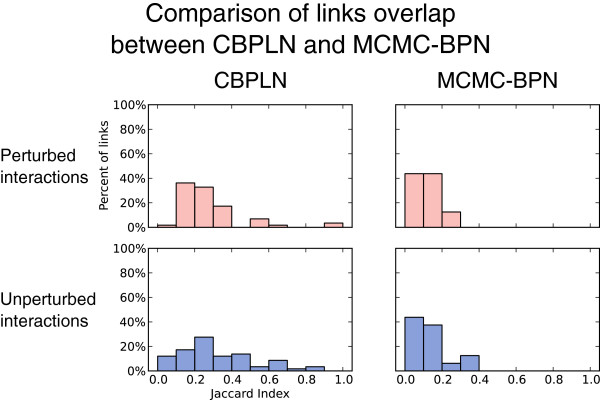
**A BPN computed for the Cirrhosis contrast.** Representations by nodes, edges, and colors are as described in Figure 3.

The three consistent BPNs in the Cirrhosis contrast were composed entirely of immune response-related processes, as shown in Figure 5. While we anticipated seeing a response in terms of liver-related processes as in the two previous analyses, two factors likely played a large role in the dominance of the immunity processes. First, all cirrhosis patients had sustained infection by HCV. Second, samples in the previous two analyses contained RNA extracted solely from hepatocytes, the cells responsible for the bulk of metabolic functions of the liver. The samples in this contrast (as well as Very Advanced HCC) were from the whole liver. Thus, they contained a mixture of cell types, which could dilute the signal from metabolic processes. Our results corroborate those found by Wurmbach *et al.*[18], who categorized the bulk of the differentiated genes as participating in immune response.

#### Very Advanced HCC

**Figure 6:**
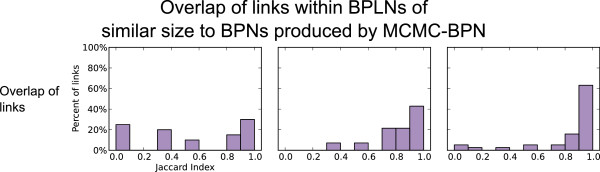
**A BPN computed for the Very Advanced HCC contrast.** Representations by nodes, edges, and colors are the same as described in Figure 3.

The majority of processes in the three similar BPNs of the Very Advanced HCC contrast related to cell replication, owing to the advanced nature of HCC in the patients from whom the samples were derived. (See Figure 6.) The largest component of the BPN contained 17 processes and 18 links, including both down- and up-regulated processes, largely including processes related to cell replication. The BPN contained a few links related to liver-specific functions, however, such as that between KEGG_VALINE_LEUCINE_AND _ISOLEUCINE_DEGRADATION and OXIDOREDUC TASE_ACTIVITY_ACTING_ON_THE_CH_CH_GROUP_OF_DONORS, both down-regulated in comparison to control patients, indicating the progression of liver damage in the HCC patients. Interestingly, REACTOME _INNATE_IMMUNITY_SIGNALING was down-regulated in HCC patients compared to controls, suggesting a breakdown in immune response. MCMC-BPN reported a significant link between this process and REGULATION_OF_MITOTIC_CELL_CYCLE.

### Comparison with CBPLN

We compared performance of MCMC-BPN to the CBPLN method by running MCMC-BPN over the day 8 CS vs. HM dataset taken directly from the CBPLN study [15], which featured the same gene expression and interaction data as the CS vs. HM study presented above, but a subset of older annotation data from MSigDB. Specifically, the CBPLN study featured a set of 18 processes significantly upregulated in CS in comparison to HM that we had manually identified and selected.

We performed five independent runs of MCMC-BPN on the CBPLN day 8 dataset. Two runs had identical sets of links with values of *α* and *β* between 0.30 and 0.35. The other three runs had high values of 0.60 and 0.65 for both *α* and *β*. We retained only the BPNs in the first group for further analyses. Both these BPNs contained 14 of the 18 terms and 16 (10.7%) of the 150 possible links, which explained 1,028 interactions, including 719 (59.7%) of the 1,205 perturbed interactions.

CBPLN produces a BPN with directed links. We ignored these directions to facilitate comparison to MCMC-BPN. We considered a link significant in the CBPLN results if the corrected p-value was at most 0.01, per the original CBPLN study [15]. The resulting undirected BPN for CBPLN contained all 18 processes with 58 links (38.7% of all possible links). The links explained 2,103 interactions, including 1,125 perturbed interactions (93.4% of all perturbed interactions).

**Figure 7:**
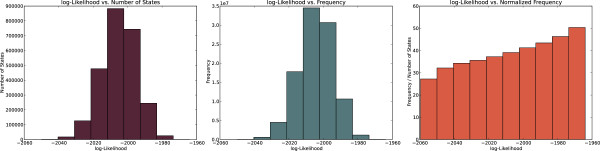
**Comparison of overlap of links between BPNs produced by CBPLN and MCMC-BPN.** Representation by axes and bar heights are as described in Figure 2.

Compared to the BPN produced by CBPLN, the BPNs produced by MCMC-BPN explained approximately two-thirds (63.9%) as many perturbed interactions in the underlying response network, however, they incorporated only approximately one-quarter (27.6%) as many links as the BPN produced by CBPLN. As shown in Figure 7. The links in BPNs from MCMC-BPN had much less overlap (81.3% of links with a maximum JI at most 0.2 for all interactions, and 87.5% for perturbed interactions) when compared to the links in the BPN produced by CBPLN (39.7% for all interactions and 37.9% for perturbed interactions).

Thus, while MCMC-BPN produced BPNs which explained somewhat less of the response network than the BPN produced by CBPLN, it did so using a much more concise, much less redundant set of links. Furthermore, CBPLN required explicitly defining the set of links for which to test for significant perturbation, whereas MCMC-BPN did not require any such specification.

Finally and most importantly, we note that MCMC-BPN was able to compute all five BPNs in fewer than 40 hours cumulative runtime on a standard modern desktop PC, whereas CBPLN required several hundred hours of cumulative runtime on a high-performance computing cluster on the same dataset, primarily due to its need to build empirical distributions to determine the statistical significance of each link. Executing CBPLN becomes nearly intractable on the full CS vs. HM, Cirrhosis, and Very Advanced HCC datasets. As stated in the “ Motivation” section, the computational expense of running CBPLN to compute links between more than a few dozen processes served as one of our primary motivations for developing MCMC-BPN.

### Comparison with BPLNs

We also compared MCMC-BPN to the BPLN method presented by Dotan-Cohen *et al.*[13]. We computed BPLNs for the three contrasts using the method of Dotan-Cohen *et al.*[13] (see the section titled “Computation of BPLNs”). We used the same input annotations as for MCMC-BPN runs, i.e., those processes found significantly perturbed for the CS vs. HM contrast by GSEA. Since BPLN does not consider the state of perturbation of genes in the interaction network, we restricted the interaction network to all the perturbed interactions. Like CBPLN, BPLN also produced directed links between processes, so we considered a significant link in either direction sufficient to indicate a significant undirected link.

For each contrast and for each of two stringent significance thresholds, Table 3 lists the number of significant (undirected) links in the BPLN, the number of processes connected by these links, and the number of interactions that these links explain. The first significance threshold of 0.0001 is an arbitrary albeit reasonable threshold that an investigator might select when exploring results from BPLN. The second threshold produces a BPLN with a number of links as close to, but no fewer than, the number of average links reported for MCMC-BPN (shown in the final row for the contrast). We discuss these results below but only for the second threshold for each contrast, in order to avoid repetitiousness.

**Table 3:** Statistics on BPLNs computed for the CS vs. HM contrast

**Link** **significance** **threshold**	**Processes**	**Links**	**Explained** **perturbed** **interactions**	**Unexplained** **perturbed** **interactions**
**CS vs. HM**				
0.0001	191	1049	3186	675
1.51 ×10^−25^	23	20	698	3163
MCMC-BPN	27.6	20.0	1686.0	2175.0
**Cirrhosis**				
0.0001	99	199	994	960
9.04 ×10^−14^	15	14	176	1778
MCMC-BPN	18.0	14.0	380.0	1574.0
**Very Advanced HCC**				
0.0001	307	7249	13213	2187
2.26 ×10^−81^	27	38	2776	12624
MCMC-BPN	44.0	36.7	5670.7	9729.3

“Link significance threshold” indicates the threshold value for which a link’s Benjamini-Hochberg-corrected p-value must be less than or equal to in order to declare the link significant. “Processes” indicates the number of processes involved in the BPLN and “links” the number of significant links between those processes. “Explained perturbed interactions” indicates the number of perturbed interactions explained by the links in the BPLN. “Unexplained perturbed interactions” indicates the number of perturbed interactions that are not explained by any link in the BPLN.

#### CS vs. HM

**Figure 8:**
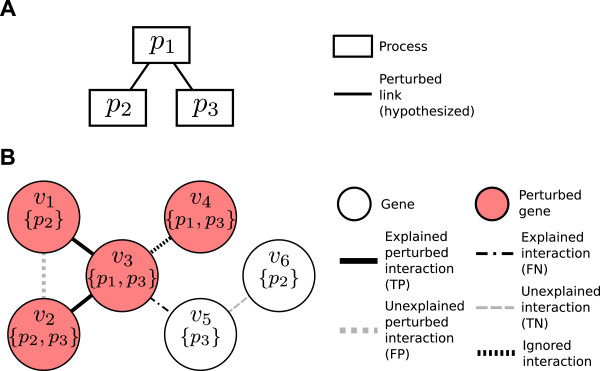
**Redundancy of links in BPLNs of similar sizes to BPNs of corresponding contrasts.** Representation by axes and bar heights are as described in Figure 2, except that all interactions are perturbed.

The BPLN produced at the cutoff of 1.51 ×10^−25^ gave the same number of significant links as the MCMC-BPN runs (20), but the BPLN links explained only 41% of the perturbed interactions explained by MCMC-BPN (see Table 3). Further, 45% of the links in the BPLN at this cutoff had a maximum JI between 0.8 and 1 (see Figure 8 (left)).

#### Cirrhosis

A threshold of 9.04 ×10^−14^ gave a number of significant links for BPLN that matched the reported average for MCMC-BPN. At this threshold, the links in the BPLN results explained fewer than half the number of perturbed gene-gene interactions. Over 65% of links had a maximum JI of 0.8 or greater (see Figure 8 (center)).

#### Very Advanced HCC

At the threshold of 2.26 ×10^−81^, the BPLN contained 38 significant links, matching as closely as possible to the reported average of 36.7 for MCMC-BPN. These links explained less than half the number of perturbed interactions as those explained by the links from MCMC-BPN. The links from BPLN involved fewer processes overall compared to MCMC-BPN. The links from the BPLN at this threshold, however, displayed a large amount of redundancy, with 88% having a maximum JI of 0.8 or greater; see Figure 8 (right).

Thus, for all contrasts, the links reported by BPLN proved less informative and more redundant than those reported by MCMC-BPN. We concluded that BPLN computed a much poorer summary of the perturbed gene interaction network in comparison to MCMC-BPN.

### Behavior of the MCMC

Since our Markov Chain has the property of irreducibility (the MCMC can reach all states from any given state with positive probability) and aperiodicity (the MCMC will not remain trapped in cycles), we expect that, given sufficient number of steps, MCMC will visit each state in the solution space with a frequency proportional to the likelihood of the state [27]. To demonstrate that MCMC-BPN follows this behavior, we performed five additional runs of MCMC-BPN for the CS vs. HM contrast wherein we recorded the frequency with which the MCMC visited each state. In these runs, we fixed the parameters to the most probable values as determined by the first five runs (*λ* = 0.01, *α* = 0.2, *β* = 0.35), and permitted only links-based transitions.

**Figure 9:**
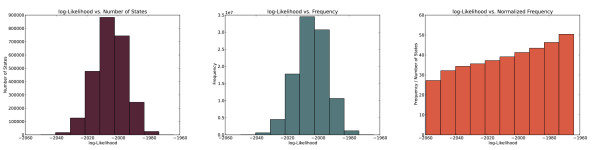
**Distributions of states and visitation frequencies.** For all plots, the *x*-axis corresponds to the logarithm of the likelihood of a state. The *y*-axes correspond to (a) the number of states, (b) frequency of visiting states with each log-likelihood value, and (c) frequency of visiting states divided by the number of states at that likelihood.

The plot in Figure 9 (left) shows the distribution of the number of distinct states visited with a given likelihood, for different values of the likelihood. The plot in Figure 9 (center) shows the distribution of the likelihoods in terms of the total number of times a state with a given likelihood was visited. Together, these plots indicate that the MCMC visited the most abundant states the most frequently. When we normalized the total number of visits by the number of distinct states, however, we observed that the MCMC visited the most likely states more frequently than those with lower likelihoods, as shown in Figure 9 (right). We observed very similar behavior in all five runs for recording the state frequencies. These results suggest that the stationary distribution of our Markov Chain is indeed one where the probability of visiting a state is proportional to its likelihood.

## Conclusions

We have presented a method for computing connections between biological processes specific to a biological context corresponding to comparing gene expression measurements from two conditions (e.g., case-control gene expression studies). Our method, which we call MCMC-BPN, uses MCMC to search a solution space of possible inter-process links that can explain the perturbed interactions between genes of the processes. We computed BPNs for three liver-related contrasts: (i) rat hepatocytes in CS compared to those in HM (CS vs. HM), and samples from livers of human patients with (ii) HCV-induced cirrhosis (Cirrhosis) or (iii) HCC (Very Advanced HCC) compared to samples from patients with healthy livers.

The BPNs varied in size, roughly in proportion to the number of perturbed interactions for the contrast. The BPNs explained around 20–40% of the perturbed interactions per contrast, using only 0.1% of the possible links, and exhibited very little redundancy. In contrast, BPNs computed by CBPLN and BPLN contained several more links than MCMC-BPNs. Moreover, these methods produced BPNs with considerable redundancy. We demonstrated that the BPNs reported contextually relevant connections between processes, such as cell-growth processes related to late-stage cancer in the Very Advanced HCC contrast, or metabolic pathway processes related to better retention of physiological function in CS vs. HM.

In the Cirrhosis and Very Advanced HCC contrasts, we observed a bifurcation in the BPNs reported by MCMC-BPN, where one set contained BPNs that explained many more perturbed interactions than the other. We noticed that during these runs, the poorer quality BPNs tended to assume high values for the false-negative rates, suggesting that the MCMC entered a solution space of poor likelihood that nevertheless was large and distant from more optimal solution spaces. One possible solution to alleviating this problem is to monitor the MCMCs as they progress, terminating or restarting those that diverge greatly from solutions of high likelihood. A more simple solution would be to restrict the values which the parameters can take even further (e.g., allow a maximum value of 0.7 rather than 0.95 for the false-negative parameter).

It may be possible to increase both the number of probable links (and thus the connectivity within a BPN), as well as the probabilities for each link that contributes strongly to the overall likelihood by calculating the link probability as the fraction of most likely BPNs (e.g., the top 100,000) in which the link appears. Strategies to reduce the required number of MCMC steps, and thus the running time, include pre-computing the possible contribution of each link to the likelihood before starting the MCMC, and pruning out the links with low contribution, thus significantly reducing the search space. We hope to include these and other improvements in future versions of MCMC-BPN. Alternatively, state-space-searching algorithms other than MCMC could be applied, such as simulated annealing (SA). We believe that MCMC-BPN and future extensions will prove useful in revealing the larger stories hidden within the ever-increasing amounts of high-throughput life science data.

## Methods

### Computing gene expression perturbation

For each contrast, we applied Linear Models for Microarray Data (LIMMA) [6] to the microarray data to compute p-values indicating the significance of differential expression of each gene. We declared all genes with a LIMMA *p*-value≤0.05 as *perturbed*.

### Selection of processes for computation of BPNs

The number of candidate BPNs is O2n2, where *n* is the number of processes. Thus, the space of candidate BPNs grows extremely rapidly in comparison to the number of processes considered. For this reason, prior to running MCMC-BPN, we screened the processes to include only those that showed significant perturbation as determined by GSEA [7]. For each contrast, we retained any processes with a false discovery rate (q-value) at or below the threshold value of 0.1. We also excluded those processes with fewer than 10 genes or more than 300 genes, to remove overly-specific and overly-general processes, respectively.

### The MCMC-BPN algorithm

#### Identifying perturbed cross-annotated interactions

Let *G*(*V*,*E*) be an undirected graph where *V* is the set of genes and *E*={(*u*,*v*), *u*,*v*∈*V*, *u*≠*v*,} the set of interactions. Let *P* be the set of all processes annotating one or more genes in *V*. We denote the set of processes annotating a specific gene *v* as *P*_*v*_⊆*P*. For a pair of processes *p*_*i*_,*p*_*j*_∈*P*, *p*_*i*_≠*p*_*j*_, we define *C*_*ij*_, the set of interactions *cross-annotated* by *p*_*i*_ and *p*_*j*_, to be those interactions where one incident gene is annotated by term *p*_*i*_, the other gene is annotated by term *p*_*j*_, but both genes are not annotated by both *p*_*i*_ and *p*_*j*_. In other words,

$$C_{\text{ij}}=\left\{(,u,,,v,),\in ,E,|,p_{i},\in ,P_{u},,,\;,p_{j},\in ,P_{v},,,\;,\{,p_{i},,,p_{j},\},⊈,P_{u},\cap ,P_{v}\right\}.$$

**Figure 10:**
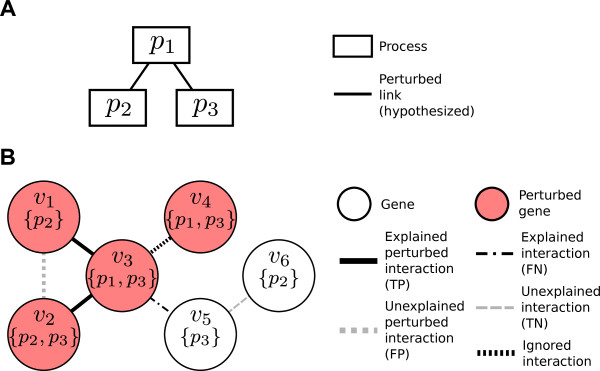
**An example interaction network and Bayesian network model.** A possible BPN **(A)** to explain the perturbation in the underlying gene-gene interaction network **(B)**. In the BPN **(A)**, nodes represent processes, while edges between nodes represent hypothesized perturbed links between the incident processes. In the interaction network **(B)**, nodes represent genes, where red coloration indicates perturbation, and sub-labels enclosed in parentheses represent processes to which each gene belongs. Edges represent interactions between those genes, and bold edges indicate a perturbed interaction. Abbreviations: TP: true positive; FP: false positive; FN: false negative; TN: true negative.

For example, in Figure 10, *C*_1,3_={(*v*_2_,*v*_3_),(*v*_3_,*v*_5_)}. Note that it does not include (*v*_3_,*v*_4_) because both genes belong to processes *p*_1_ and *p*_3_. We use *C* to denote the set of all cross-annotated interactions, i.e.,

$$C=\underset{p_{i},p_{j}\in P,\;p_{i}\neq p_{j}}{⋃}C_{\text{ij}}.$$

We then define the *perturbed cross-annotated interactionsD*⊆*C* as the subset of cross-annotated interactions for which both incident genes are perturbed. Lastly, we denote the subset of perturbed interactions cross-annotated by specific terms (*p*_*i*_,*p*_*j*_), where *p*_*i*_≠*p*_*j*_, as *D*_*ij*_=*C*_*ij*_∩*D*. For example, in Figure 10, *D*_1,3_={(*v*_2_,*v*_3_)}.

#### Calculation of BPN likelihood

Let *L* be the set of all possible links, comprised of all unordered pairs of processes (*p*_*i*_,*p*_*j*_)∈*P*. We say a link (*p*_*i*_,*p*_*j*_)*explains* the interactions which its terms cross-annotate, i.e., *C*_*ij*_. Our method aims to find the smallest set *X* of links that explains as many perturbed interactions in *D* as possible, while explaining as few unperturbed interactions in *C*∖*D* as possible. To gauge how well *X* explains *D*, we formulate a likelihood function

Pr(X,D)=Pr(X)Pr(D|X)

composed of the following terms: 

● Pr(*X*): the probability of selecting this subset of links *X* from among all possible pairs of terms in *P*

● Pr(*D*|*X*): the probability that the links in *X* explain the observed perturbed interactions *D*

We explicitly define each of these individual probabilities below.

We calculate the probability Pr(*X*) as a Bernoulli distribution:

$$\;\mathrm{Pr}(X)=\lambda^{\left|X\right|}{\left(1-\lambda \right)}^{\left|L-X\right|},$$

where 0≤*λ*≤1 represents the prior probability of selecting a given link from the set of all links.

We introduce additional terminology to define Pr(*D*|*X*), the second probability in the likelihood function. We can categorize each interaction within *C* among four classes, depending on whether or not the interaction is in *D* (the set of perturbed interactions), and whether or not the interaction is explained by a link in *X*. We define four sets of edges *I*_*oh*_⊆*C*, where *o*,*h*∈{0,1}. Here, let *o* = 1 if and only if the interaction is perturbed (i.e., in *D*), and *h* = 1 if and only if one or more links in *X* explains the interaction. We list the four subsets below and list members of each set in the example given in Figure 10: 

(i) *I*_11_: perturbed interactions explained by at least one link in *X*, i.e., the “true positives” (*I*_11_ = {(*v*_1_,*v*_3_),(*v*_2_,*v*_3_)} in the example);

(ii) *I*_10_: perturbed interactions not explained by any links in *X*, i.e., the “false positives” (*I*_10_ = {(*v*_1_,*v*_2_)} in the example);

(iii) *I*_01_: interactions that are not perturbed, but which are explained by one or more links in *X*, i.e., the “false negatives” (*I*_01_ = {(*v*_3_,*v*_5_)} in the example);

(iv) *I*_00_: interactions that are not perturbed and are not explained by any links in *X*, i.e., the “true negatives” (*I*_00_ = {(*v*_5_,*v*_6_)} in the example).

We briefly note here that the example in Figure 10 contains one additional interaction, (*v*_3_,*v*_4_), excluded from consideration in these categories as it does not meet the definition of a cross-annotated interaction, since both *v*_3_ and *v*_4_ have identical annotations.

With this notation, we define the probability Pr(*D*|*X*) as the following combination of Bernoulli distributions:

$$\mathrm{Pr}(D|X)=\alpha^{\left|I_{10}\right|}{\left(1-\alpha \right)}^{\left|I_{00}\right|}\beta^{\left|I_{01}\right|}{\left(1-\beta \right)}^{\left|I_{11}\right|}$$

where *α* represents the false-positive rate (i.e., the prior probability that a perturbed interaction is not explained by any link in *X*), and *β* represents the false-negative rate (i.e., the prior probability an unperturbed interaction is explained by one or more links in *X*).

Since we do not know *a priori* which values for the parameters *λ*, *α*, and *β* will lead to the greatest likelihood, we attempt to learn estimates for these parameters as well. We denote a particular configuration of parameters as *Φ*(*λ*,*α*,*β*). We define the likelihood for a configuration of parameters *Φ* and links *X* as

$$\begin{array}{l} \mathrm{Pr}(\Phi ,X,D)=\mathrm{Pr}(\Phi )\mathrm{Pr}(X|\Phi )\mathrm{Pr}(D|\Phi ,X)\\ \;\propto \lambda^{\left|X\right|}{\left(1-\lambda \right)}^{\left|L-X\right|}\alpha^{\left|I_{10}\right|}{\left(1\;-\alpha \right)}^{\left|I_{00}\right|}\beta^{\left|I_{01}\right|}{\left(1\;-\beta \right)}^{\left|I_{11}\right|}, \end{array}$$

since we assume that all configurations of parameter values are equally likely.

#### Distributions of the parameters

We restrict the values of the parameters to discrete, rather than continuous, distributions. We allow *λ*, the prior probability of selecting a link, to take values in the set {0.05k∣1≤k≤10,k∈ℕ}∪{0.00001,0.00005,0.0001,0.0005,0.001,0.005,0.01}. This set spans a wide enough range to cover computing BPNs from many possible links, where only a small fraction may be included, to few links, where a large fraction of the links may be included. We restrict the set of values for both *α*, the false-positive rate, and *β*, the false-negative rate, to {0.05k∣1≤k<20,k∈ℕ}.

#### Design of the Markov chain

We define the set of all possible states in the Markov chain as ℳ. Each state m(Φ,X)∈ℳ consists of two configurations: a configuration of parameters *Φ* and a set of explanatory links *X*. We restrict each state *m*(*Φ*,*X*) to having two types of neighboring states *m*^′^(*Φ*^′^,*X*^′^): neighbors with different parameter configurations, i.e., *Φ*^′^≠*Φ* but *X*^′^=*X*, and neighbors with different links configurations, i.e., *Φ*^′^=*Φ* but *X*^′^≠*X*. We restrict the parameters-based neighbors of *m*(*Φ*,*X*) to be those with a different value for only one parameter. We restrict the links-based neighbors of *m*(*Φ*,*X*) to be those with a set of links containing one additional or one fewer link than *X*^′^.

#### Design of the Markov chain Monte Carlo

In order to transition from the current state *m*(*Φ*,*X*) to a neighboring state *m*^′^(*Φ*^′^,*X*^′^), we propose a links-based neighbor with probability *ρ* and a parameters-based neighbor with probability 1−*ρ*, where *ρ*, 0≤*ρ*≤1. If we propose a links-based neighbor, then we draw neighbor *m*^′^(*Φ*^′^=*Φ*,*X*^′^≠*X*) uniformly at random from the set of all links-based neighbors of *m*(*Φ*,*X*). Otherwise, we draw a neighbor *m*^′^(*Φ*^′^≠*Φ*,*X*^′^=*X*) uniformly at random from the set of all parameters-based neighbors. In this study, we set *ρ*=0.9, so that approximately 90% of proposed transitions were links-based.

Once we select a neighbor for a proposed transition, we follow the Metropolis-Hastings algorithm for MCMC, i.e., we accept the transition from *m*(*Φ*,*X*) to *m*^′^(*Φ*^′^,*X*^′^) with probability

$$P_{\text{accept}}=\min\left(1,\frac{\mathrm{Pr}(\Phi^{\prime },X^{\prime },D)N(m(\Phi ,X))}{\mathrm{Pr}(\Phi ,X,D)N(m^{\prime }(\Phi^{\prime },X^{\prime }))}\right),$$

where *N*(*m*(*Φ*,*X*)) is the number of neighbors of state *m*(*Φ*,*X*) and *N*(*m*^′^(*Φ*^′^,*X*^′^)) is the number of neighboring states of state *m*^′^(*Φ*^′^,*X*^′^). We note that because of the design of the Markov chain, *N*(*m*(*Φ*,*X*)) and *N*(*m*^′^(*Φ*^′^,*X*^′^)) are equal and thus cancel each other out. If we accept the transition, then *m*^′^(*Φ*^′^,*X*^′^) becomes the current state; otherwise, *m*(*Φ*,*X*) remains the current state. Note that any time the proposed state has a greater likelihood than the current state, we will accept the transition. On the other hand, we still accept transitions to proposed states with poorer likelihoods with probability proportional to the ratio of likelihood of the proposed state to the likelihood of the current state. By allowing unfavorable transitions, the MCMC may escape local minima.

At the start of each MCMC run, we begin at a state which includes no links (i.e., *X*=*∅*) and where each parameter is set to a value drawn uniformly at random from its respective set of possible values. We then allow the MCMC to progress for a designated number of steps, as described below.

#### Reporting the BPN

We run the MCMC for a burn-in period of 10^7^ steps. Following the burn-in period, we run the MCMC for 10^8^ steps, recording at each step the value of each parameter *λ*, *α*, and *β*, as well as the links in *X*. Finally, the probability of a parameter (*λ*, *α*, or *β*) being a particular value is the fraction of recorded steps in which the parameter was observed at this value. The probability of a link Pr(*l*) is the fraction of recorded steps in which a given link *l*∈*L* was observed in *X*. We reported links with probabilities meeting or exceeding a user-defined threshold *θ* (we used *θ*=0.7) as those comprising the BPN, i.e., the reported BPN is the set of links {*l*∈*L*∣ Pr(*l*)≥*θ*}.

### Computation of BPLNs

For each experiment, using significantly perturbed processes as determined by GSEA and the subnetwork induced by the set of perturbed interactions *D* as the inputs, we computed BPLNs as described by Dotan-Cohen *et al.*[13]. Briefly, to test whether a link exists from one process to another, BPLN counts the number of genes belonging to the second process that also neighbor genes in the first process. Using a one-sided Fisher’s Exact Test, it then determines whether this count is greater than expected by chance. After applying Benjamini-Hochberg correction for multiple hypothesis testing [28], we declared significant and included all links which had a *q*-value≤0.05. Since links in BLPN are directed, and links in BPNs returned by the MCMC method are undirected, we considered two processes *p*_*i*_,*p*_*j*_∈*P* to be linked in the BPLN if the *q*-value for either (*p*_*i*_,*p*_*j*_) or (*p*_*j*_,*p*_*i*_) was significant.

### Measuring redundancy within a BPN

#### Redundancy of links

To assess the redundancy of links in a BPN, we calculated the JI of the sets of interactions cross-annotated by every pair of links in the BPN. We calculated the JI on the basis of only perturbed interactions and only unperturbed interactions. For each link, we recorded the maximum JI between that link and all other links. We computed the mean of each of the JIs for each link over the BPNs computed for a contrast by independent executions of MCMC.

#### Explaining links per interaction

For each interaction explained by one or more links in a BPN, we counted the number of links that explain it. For every positive integer, *k*, we recorded the fraction of interactions which that were explained by *k* links. We reported the average fraction over the BPNs computed for a contrast.

### Software availability

Our Python implementations of MCMC-BPN, CBPLN, and BPLN are available under the Open Source Initiative-approved MIT License from the Python Package Index at http://pypi.python.org/pypi/BiologicalProcessNetworks.

## Competing interests

The authors declare that they have no competing interests.

## Authors’ contributions

CDL, PR, and TMM designed the experiments. CDL and TMM designed the MCMC-BPN algorithm. CDL implemented the software. CDL, PR, and TMM provided the biological interpretations. All authors read and approved the final manuscript.

## Supplementary Material

Additional file 1 **Supplementary information.** A PDF file containing (a) details on how we executed the MCMC-BPN software to obtain and visualize our results and (b) a description of the files in the supplementary results. Click here for file

Additional file 2 **Supplementary results.** A zipped file containing all the five BPNs for each of the contrasts studied and the parameters estimated by each run of the software. Click here for file
