# Supplementary material for: Summarizing cellular responses as biological process networks
Source: BMC Syst Biol. 2013 Jul 29;7:68. doi: 10.1186/1752-0509-7-68 (PMC3751784; doi:10.1186/1752-0509-7-68)
Supplement: Additional file 1 — Supplementary information. A PDF file containing (a) details on how we executed the MCMC-BPN software to obtain and visualize our results and (b) a description of the files in the supplementary results. [file 1752-0509-7-68-S1.pdf]

Supplementary Information
Summarizing Cellular Responses as Biological Process Networks

Christopher D. Lasher[1], Padmavathy Rajagopalan[2,3], and T M Murali[3,4,*]

[1]Genetics, Bioinformatics, and Computational Biology PhD Program
[2]Department of Chemical Engineering
[3]ICTAS Center for Systems Biology of Engineered Tissues
[4]Department of Computer Science, Virginia Tech, Blacksburg, VA 24061 USA

Email: Christopher D Lasher - lasher@vt.edu; Padmavathy Rajagopalan -padmar@vt.edu; T M Murali*- murali@cs.vt.edu;

*Corresponding author

1 Program Usage Notes

1.1 Execution of `mcmcbpn`

We ran the program `mcmcbpn` five times independently for each dataset. We executed each run of `mcmcbpn` with the following options:

- `--burn-in=10000000`
- `--steps=100000000`
- `--disable-swaps`
- `--fixed-distributions`
- `--free-parameters`
- `--bzip2`

1.2 Rendering the BPNs

To render the networks, we used the script `render_bpln_graph.py` with the following options:

- `-g` with the appropriate GSEA results file
- `--probability`
- `-s 0.7`
- `--annotation1-col term1`
- `--annotation2-col term2`
- `--significance-col probability`

1.3 Calculating summary statistics for the BPN and BPLN results

To calculate the summary statistics for the BPN and BPLN results, we used the script `summarize_bpns.py` with the following options:

- `--links-cutoff=` the appropriate threshold value for significance (e.g., `0.7`)
- `--links-lt` (BPLN results only)
- `--expression-file=` with the appropriate gene expression data file

2 Description of Supplementary Results Files

Here we describe the contents of the files contained in the directory `supplementary-results` obtained by unzipping the second additional file `supplementary-results.zip`. The directory is divided into four subdirectories. Three of these subdirectories (`cirrhosis`, `cs_vs_hm`, and `very_advanced`), contain results for the respective experiments described in the main publication. Within each of these subdirectories exist three types of plain-text tab-separated values (TSV) files:

Links results one file per each of the five independent runs of MCMCBPN for the given experiment, containing the probabilities each link should be included in the final BPN. The column descriptions are as follows:

column	description
term1	the first of the two biological processes in the link
term2	the second of the two biological processes in the link
probability	the proportion of steps in which the link was included in the BPN, representing the probability the link should be included in the final BPN

Parameters results one file per each of the five independent runs of MCMCBPN for the given experiment, containing the probabilities parameters should be at a given value in order to maximize the likelihood of the BPN. The column descriptions are as follows:

column	description
parameter	the parameter; "link_prior" represents λ, "link_false_positive" represents α, "link_false_negative" represents β (see Methods)
value	a particular value assumed for the given parameter
probability	the proportion of steps in which the given parameter assumed the given value

BPLN results the significance of links as calculated by BPLN [1] (see Methods). The column descriptions are as follows:

column	description
term1	the first of the two biological processes in the link
term2	the second of the two biological processes in the link
fishers_exact	the uncorrected p-value for the significance of the link (see Methods)
bh_corrected_p_value	the Benjamini-Hochberg [2] corrected q-value for the significance of the link (see Methods)

The final directory, `cbpln_comparison`, contains the links and parameters results files for the MCMC-BPN runs on the original CBPLN study data for day 8 [3], as well as the results file for CBPLN from the original study. The CPBLN file has the following format:

column	description
term1	the first of the two biological processes in the link
term2	the second of the two biological processes in the link
bh_corrected_p_value	the Benjamini-Hochberg [2] corrected q-value for the significance of the link (see Methods)

References

1. Dotan-Cohen D, Letovsky S, Melkman AA, Kasif S: **Biological Process Linkage Networks**. *PLoS ONE* 2009, **4**(4):e5313.

2. Benjamini Y, Hochberg Y: **Controlling the False Discovery Rate: A Practical and Powerful Approach to Multiple Testing**. *Journal of the Royal Statistical Society. Series B (Methodological)* 1995, **57**:289–300.

3. Lasher CD, Rajagopalan P, Murali TM: **Discovering Networks of Perturbed Biological Processes in Hepatocyte Cultures**. *PLoS ONE* 2011, **6**:e15247.
